# *GSTM1*, *GSTT1*, and *GSTP1* polymorphisms and colorectal cancer risk in Polish nonsmokers

**DOI:** 10.18632/oncotarget.25031

**Published:** 2018-04-20

**Authors:** Justyna Klusek, Anna Nasierowska-Guttmejer, Artur Kowalik, Iwona Wawrzycka, Piotr Lewitowicz, Magdalena Chrapek, Stanisław Głuszek

**Affiliations:** ^1^ Department of Surgery and Surgical Nursery with a Research Laboratory, Faculty of Medicine and Health Sciences, Jan Kochanowski University, Kielce, Poland; ^2^ Department of Pathology, Faculty of Medicine and Health Sciences, Jan Kochanowski University, Kielce, Poland; ^3^ Department of Molecular Diagnostics, Holy Cross Cancer Centre, Kielce, Poland; ^4^ Department of General, Oncological and Endocrinological Surgery, Voivodeship Hospital, Kielce, Poland; ^5^ Department of Probability Calculus and Statistics, Institute of Mathematics, Jan Kochanowski University, Kielce, Poland

**Keywords:** colorectal cancer, GSTT1, GSTM1, GSTP1, gene polymorphism

## Abstract

Glutathione S-transferase (GST) enzymes are responsible for cellular detoxification of many carcinogens and are important anticancer elements. This study assessed potential relationships between *GSTM1*, *GSTT1*, and *GSTP1* polymorphisms and colorectal cancer (CRC) risk in Polish nonsmokers. We also analyzed the influence of *GST* gene polymorphisms on CRC clinical and histopathological features. Our study included 197 CRC patients and 104 healthy controls. *GSTM1*, *GSTT1*, and *GSTP1* polymorphisms were evaluated using qPCR. Polymorphism frequencies observed in our control group corresponded to those in other European populations. The *GSTM1 null* and *GSTT1 null* genotypes were observed with similar frequencies in both CRC patients and controls (*GSTM1 null*: 46.7% vs. 45.2%; *GSTT1 null*: 15.7% vs. 20.2%). *GSTP1 Ile/Ile*, *Ile/Val*, and *Val/Val* genotype frequencies were respectively 42.1%, 48.2%, and 9.6% in patients and 48.1%, 42.3%, and 9.6% in controls. *GSTT1* polymorphism correlated with higher tumor grade in CRC patients, and the *GSTM1 null/null* genotype was associated with more frequent metastasis to lymph nodes (pN classification). Our results suggest that *GST* gene polymorphisms may influence CRC tumor grade and stage.

## INTRODUCTION

In Poland in 2013, colorectal cancers (CRC) were the third most common cancers in males (8,726 cases, 11.2% of all cases) and the second most common in females (7,173 cases, 9.2% of all cases). A total of 16,000 new CRC cases were diagnosed in 2013, with deaths reported in 10,562 of these cases (5,851 men, second most common cause of cancer-related death; 4,711 women, third most common cause of cancer-related death) [1]. Globally, CRC was the third most common cancer among both males and females in 2012, occurring in 1.4 million patients [1].

CRC causes include heterogeneous, controllable, and external factors related to lifestyle, such as diet and socioeconomic standing [2]. Such factors include high consumption of red meat, insufficient intake of fiber, vitamins, and minerals (calcium, selenium, zinc, copper, manganese, folic acid), excessive fat consumption, obesity, and lack of exercise. Diets contributing to chronic inflammation are a cause of CRC. An extensive meta-analysis conducted in 2017 based on eight studies, 103 articles, and approximately 900,000 cases assessed the risk of CRC development in relation to the inflammatory dietary index (DII). CRC incidence corresponds to a high DII score (indicating a pro-inflammatory diet). High DII scores are associated with consumption of red meat and high-fat products, whereas low scores are associated with consumption of fruits, vegetables, and other low-fat foods [3]. Previous studies have also highlighted the link between processed red meat and colorectal cancer [4, 5, 6]. However, the relationship between red meat processing that produces heterocyclic amines (HCA) and polycyclic aromatic hydrocarbons (PAH) and CRC risk is still uncertain. Genetic changes, including variants of genes responsible for detoxification, are important for CRC development, [7, 8]. Glutathione S-transferases are believed to play roles in deactivating pathogenic compounds and allergens during phase II of biotransformation.

Glutathione S-transferases are a multi-gene family of enzymes that inactivate toxins in glutathione-coupling reactions. They are responsible for cellular detoxification of environmental pollutants, carcinogens, chemotherapeutics, reactive oxygen species (ROS), and a wide spectrum of xenobiotics. Enzyme activity loss or reduction inhibits toxin neutralization. Many tumor studies focus on three of the eight transferase classes, namely theta (θ), mu (μ), and pi (π), encoded by genes *GSTT1, GSTM1*, and *GSTP1*, respectively [9, 10]. Overexpression of these enzymes in enterocytes in CRC suggests a role for glutathione S-transferase π (*GSTP1*) in the degradation of carcinogens [9]. Similarly, μ transferase (*GSTM1*) expression in CRC patients was 4.8 times greater in cancer cells than in healthy tissues [8]. This is presumably an adaptive cellular response to progressive disease.

*GST* genes are highly polymorphic. Mutation frequency is relatively high in humans and varies according to geographical region and ethnic group [9, 11]. For example, *GST* mutation frequency is higher in whites than Asians [12, 13]. *GSTT1* and *GSTM1* polymorphisms mainly involve deletion. If this mutation occurs in only one allele (*GSTM1* +/− or *GSTT1* +/−), enzyme production is retained; however, homozygous deletion (*GSTM1* −/−, or *GSTT1* −/−; the *null* genotype) results in total loss of enzyme activity [11].

The functional polymorphism of the *GSTP1* gene, which reduces enzymatic activity, involves an A-G substitution in exon 5 and the conversion of isoleucine to valine at position 105 of the amino acid chain (Ile105Val) [9]. Carriers of these mutations are less able to detoxify carcinogens, which may increase cancer risk [8]. Of these three enzymes, μ-class glutathione S-transferase (*GSTM1*) appears to be the most effective at neutralizing cytotoxic and genotoxic reactive compounds [14]. Nevertheless, polymorphism of all three *GST* genes may be associated with risk of gastrointestinal cancers, including CRC. Thus, this project analyzed potential relationships between *GSTM1*, *GSTT1*, and *GSTP1* and CRC risk in a non-smoking population in one region of Poland.

## RESULTS

We genotyped 301 non-smokers of both sexes aged 38–81, including 197 patients with CRC and 104 healthy controls (Table 1). There were relatively more men than women in the patient group compared to the control group due to the higher frequency of CRC cases among men in Poland. According to the National Cancer Register, CRC occurs twice as often in men than in women in Poland [1]. However, these differences did not affect the analyzed relationships, because there were no significant differences in the frequencies of examined polymorphisms with respect to sex (Table 2).

**Table 1:** Patient and control demographics

	Patients	Controls	*p*-value
	No. 197	No. 104	
Age (years): (min–max)	35–85	35–85	
Age (years): mean (± sd)	64.5 (±8.4)	61.2 (±11.1)	
Age (years): median (Q1–Q3)	65.0 (59.0–71.0)	63.0 (52.8–69.2)	0.027
Sex:			0.005
Female	78 (39.6%)	59 (56.7%)	
Male	119 (60.4%)	45 (43.3%)	

**Table 2:** GST gene polymorphism frequencies in patients and controls

	Patients (No. 197)	Controls (No. 104)	OR [95% CI]; *p*-value	OR [95% CI]; *p*-value
			crude	adjusted^*^
*GSTM1*				
wild type	105 (53.3%)	57 (54.8%)	Reference	Reference
null/null	92 (46.7%)	47 (45.2%)	1.1 [0.7–1.7]; 0.80	1.0 [0.6–1.7]; 0.96
*GSTT1*				
Wild type	166 (84.3%)	83 (79.8%)	Reference	Reference
null/null	31 (15.7%)	21 (20.2%)	0.7 [0.4–1.4]; 0.33	0.9 [0.5–1.6]; 0.66
*GSTP1*				
Ile/Ile (wild type)	83 (42.1%)	50 (48.1%)	Reference	Reference
Ile/Val	95 (48.2%)	44 (42.3%)	1.3 [0.8–2.1]; 0.30	1.2 [0.7–2.1]; 0.40
Val/Val	19 (9.6%)	10 (9.6%)	1.1 [0.5–2.7]; 0.75	1.0 [0.4–2.5]; 0.93
*GSTP1*				
Ile/Ile (wild type)	83 (42.1%)	50 (48.1%)	Reference	Reference
Ile/Val or Val/Val	114 (57.9%)	54 (51.9%)	1.3 [0.8–2.1]; 0.32	1.2 [0.7–2.0]; 0.45

^*^for age and sex.

*GST* polymorphism frequencies in the patient and control groups are shown in Table 2. The *GSTT1 null* genotype was found in 15.7% of patient samples and 20.2% of control samples. This difference was not statistically significant (*p* = 0.66). Similarly, the *GSTM1 null* mutation frequency in patients (46.7%) did not differ from that in controls (45.2%) (*p* = 0.96) (Table 2).

*GSTP1* genotype distributions were Ile/Ile 42.1%, Ile/Val 48.2%, and Val/Val 9.6% in patients and Ile/Ile 48.1%, Ile/Val 42.3%, Val/Val 9.6% in controls (Table 2). These differences were not significant. The *GSTP1* Ile/Ile variant frequency was also compared to genotypes with at least one mutant allele (Ile/Val or Val/Val). The frequency of Ile/Val and Val/Val genotypes observed in patients (57.9%) was higher than that in controls (51.9%); however, these differences were not statistically significant (*p* = 0.45) (Table 2).

We analyzed relationships between *GST* polymorphisms and demographic characteristics (age, sex), selected clinical features (tumor location), and histopathological features (histological type, stage pTNM AJCC/UICC, and grade) (Table 3). Higher grade (G3) tumors were more frequently observed in patients with *GSTT1* polymorphism (19.4% vs. 5.4% in patients with the wild type gene, *p* = 0.035). In patients with *GSTM1* polymorphism, lymph node metastasis was associated with pN1 vs. pN2 (pN classification; *p* = 0.036), suggesting that this polymorphism may affect tumor stage. *GSTP1* polymorphisms were not associated with any analyzed features.

**Table 3:** *GST* gene polymorphism associations with selected clinical-histopathological features in patients

	GSTM1			GSTT1			GSTP1		
	null/null	wildtype	*p*-value	null/null	wildtype	*p*-value	polymorphism	wildtype	*p*-value
	No. 92	No. 105		No. 31	No. 166		No. 114	No. 83	
Age (years)	64.5 (±7.7)	64.4 (±9.0)	0.87	62.5 (±9.8)	64.8 (±8.1)	0.26	65.0 (±8.1)	63.8 (±8.8)	0.28
Sex			0.77			0.072			0.14
female	35 (38.0%)	43 (41.0%)		17 (54.8%)	61 (36.7%)		40 (35.1%)	38 (45.8%)	
male	57 (62.0%)	62 (59.0%)		14 (45.2%)	105 (63.3%)		74 (64.9%)	45 (54.2%)	
Cancer type			1.0			1.0			0.18
other	1 (1.1%)	1 (1.0%)		0 (0.0%)	2 (1.2%)		0 (0.0%)	2 (2.4%)	
adenocarcinoma	91 (98.9%)	104 (99.0%)		31 (100.0%)	164 (98.8%)		114 (100.0%)	81 (97.6%)	
Stage			0.76			0.035			0.077
G1	4 (4.3%)	5 (4.8%)		0 (0.0%)	9 (5.4%)		3 (2.6%)	6 (7.2%)	
G2	76 (82.6%)	82 (78.1%)		22 (71.0%)	136 (81.9%)		96 (84.2%)	62 (74.7%)	
G3	7 (7.6%)	8 (7.6%)		6 (19.4%)	9 (5.4%)		10 (8.8%)	5 (6.0%)	
Gx	5 (5.4%)	10 (9.5%)		3 (9.7%)	12 (7.2%)		5 (4.4%)	10 (12.0%)	
Tumor location			0.93			0.26			0.82
unknown	4 (4.3%)	4 (3.8%)		2 (6.5%)	6 (3.6%)		4 (3.5%)	4 (4.8%)	
Left side^*^	70 (76.1%)	78 (74.3%)		20 (64.5%)	128 (77.1%)		85 (74.6%)	63 (75.9%)	
Right side^*^	18 (19.6%)	23 (21.9%)		9 (29.0%)	32 (19.3%)		25 (21.9%)	16 (19.3%)	
pT			0.48			0.30			0.15
unknown	19 (20.7%)	24 (22.9%)		7 (22.6%)	36 (21.7%)		22 (19.3%)	21 (25.3%)	
pT = 1	4 (4.3%)	7 (6.7%)		2 (6.5%)	9 (5.4%)		8 (7.0%)	3 (3.6%)	
pT = 2	11 (12.0%)	9 (8.6%)		1 (3.2%)	19 (11.4%)		13 (11.4%)	7 (8.4%)	
pT = 3	42 (45.7%)	45 (42.9%)		12 (38.7%)	75 (45.2%)		50 (43.9%)	37 (44.6%)	
pT = 4	16 (17.4%)	16 (15.2%)		9 (29.0%)	23 (13.9%)		21 (18.4%)	11 (13.3%)	
pT = *in situ*	0 (0.0%)	4 (3.8%)		0 (0.0%)	4 (2.4%)		0 (0.0%)	4 (4.8%)	
pN			0.036			0.50			0.45
unknown	21 (22.8%)	32 (30.5%)		10 (32.3%)	43 (25.9%)		27 (23.7%)	26 (31.3%)	
pN = 0	34 (37.0%)	40 (38.1%)		9 (29.0%)	65 (39.2%)		44 (38.6%)	30 (36.1%)	
pN = 1, 1a, 1b	33 (35.9%)	21 (20.0%)		8 (25.8%)	46 (27.7%)		35 (30.7%)	19 (22.9%)	
pN = 2a, 2b	4 (4.3%)	12 (11.4%)		4 (12.9%)	12 (7.2%)		8 (7.0%)	8 (9.6%)	

^*^Left side: transverse colon, descending colon, sigmoidum, rectum; right side: ascending colon.

## DISCUSSION

Glutathione S-transferases are responsible for neutralizing carcinogens, including heterocyclic amines (HCAs), nitrosamines, polycyclic aromatic hydrocarbons (PAHs), and many others [9], and are important elements of cellular defense systems (including defense against carcinogenic processes). Many toxins neutralized by GST transferases enter the body via the digestive tract. HCAs, for example, are produced by the thermal processing of red meat, and the intestinal epithelium is particularly susceptible to the adverse effects of these compounds. *GST* gene polymorphisms are relatively frequent and result in reduction or total loss of enzymatic activity in the corresponding protein. Reduced detoxification capacity in cells may increase intestinal mucosa exposure to carcinogenic substances. *GST* polymorphisms have been linked to increased cancer risk [7], although results thus far have been contradictory. In a study conducted in Turkey, Gorukmez [9] found no correlation between *GSTT1* and *GSTM1* polymorphisms and an increased risk of CRC. However, this study revealed a higher frequency of the *GSTP1* Ile/Ile genotype in CRC patients than healthy patients. Another study on the Turkish population also found no correlation between *GSTP1* polymorphisms and CRC, but associated *GSTM1 null* and/or *GSTT1 null* genotypes with increased risk of rectal and transverse colon cancers [15]. A meta-analysis of 44 studies by Economopoulos [16] revealed a link between *GSTM1* gene deletion and increased CRC risk in whites, but not in Asians. An analysis of 34 studies indicated the same association for *GSTT1*, but not *GSTP1* polymorphisms [14]. However, a later meta-analysis associated the *GSTM1 null* genotype with CRC risk in Asians [14]. Thus, there is still no consensus on the nature of these relationships, and no such studies had been conducted in Poland.

Our results suggest that the *GSTM1 null* genotype frequency in the general population of Poland is similar to that in other European countries [9, 17]. In CRC patients, our results were similar to those obtained by Gorukmez in Turkish patients (46.7% and 42.2% respectively) [9]. According to Hezova [17], the *GSTM1 null* genotype is present in 40–60% of Caucasians, and the *GSTT1 null* genotype in 10–20% of Caucasians. In our study, there was no difference in the *GSTM1* deletion frequency in patients and controls. Similarly, no differences in this polymorphism were observed in Korean, English [8], or Turkish [9] populations. However, Ates [15] found that this deletion nearly doubled the risk of CRC in Spanish populations. A meta-analysis of 36 studies also revealed a relationship between the *GSTM1 null* genotype and increased CRC incidence in whites, but not other ethnic groups [18]. Different results for different populations may result from diverse environmental influences and eating habits. For example, many of the abovementioned studies do not distinguish smokers from non-smokers, which is a factor highly correlated with cancer risk [7, 13, 19].

In our study, *GSTM1* polymorphism was associated with lymph node metastasis (pN calssification; *p* = 0.036), suggesting that this gene may influence tumor stage. A meta-analysis of 36 case-control studies also detected a relationship between *GSTM1* polymorphism and tumor site [18]. However other studies found no association between the *GSTM1 null* genotype and CRC clinicopathologic features [9, 20, 21].

The *GSTT1 null* genotype was found in 20.2% of control patients, where the average frequency of this deletion in whites is 10–20% [9, 17]. The distribution of this genotype in Polish patients is thus similar to that of other European countries. As with the *GSTM1* gene, no differences in *GSTT1 null* genotype frequencies were found in CRC and control patients. Kassab [22] also found no relationship between *GSTM1* or *GSTT1* genotypes and CRC risk in the population of Tunisia. A Chinese study associated the *GSTT1 null* genotype with increased CRC risk only in men, and especially in the rectum [23]. Other studies did not differentiate the effects of these polymorphisms with respect to patient sex. Our study did observe a correlation between tumor grade G3 and *GSTT1* polymorphism (19.4% in CRC vs. 5.4% in controls; *p* = 0.035). However, Gorukmez found no relationship between this polymorphism and CRC clinical or microscopic features in a Turkish population [9]. In our study, the *GSTM1 null* and *GSTT1 null* genotypes did not increase CRC risk when present together. Ates [15] reported different results in a Turkish population, suggesting that these deletions separately and together are risk factors for CRC, although this study did not account for environmental factors when selecting patient groups.

The distribution of *GSTP1* genotypes did not differ between CRC patients and controls. The *GSTP1 Ile/Ile* genotype frequency was higher in controls (48.1%) than patients (42.1%), but this difference was not significant (*p* = 0.4). *GSTP1* Ile/Val heterozygotes were more frequently observed in patients (48.2%) than controls (42.3%) (*p* = 0.4). *GSTP1* Val/Val homozygotes were observed with the same frequency in both groups (9.6%; *p* = 0.93). Frequencies of the *GSTP1* Ile/Ile genotype and *GSTP1* genotypes with at least one mutant allele (*GSTP1* Ile/Val or *GSTP1* Val/Val) also did not differ between patients and controls. Khabaz [24] in Saudia Arabia also found no relationship between any of these three genotypes and CRC risk. Studies in Bulgaria [25] and on the population of Kashmir [26] also found no relationships between *GSTP1* genotypes and CRC. However, Gorukmez [9] showed that the *GSTP1* Ile/Ile genotype occurred more often in controls than in patients. Vlaykova [25] also reported a non-significant protective role for the Val allele.

Carcinogen exposure varies according to geographic region, environmental pollution levels, and dietary habits. Similarly, frequencies of the polymorphisms evaluated here appear to differ based on patient ethnicity. These factors likely contribute to highly contradictory findings with respect to *GST* gene polymorphism-cancer associations. However, the *GSTM1 null, GSTT1 null*, and *GSTP1 Ile_105_Val* genotypes are associated with higher levels of DNA damage [23, 27]. These mutations reduce the activity of their respective enzymes, impairing cellular metabolism of carcinogenic and oxidative stress products. Thus, while CRC is diet- and lifestyle-dependent, with 85% of cases being caused by external factors [28], disease incidence is also likely dependent on genetic factors.

In some cases, decreased activity of one *GST* enzyme class may be compensated for by the presence or increased expression of an enzyme of another class [13]. One study of nearly 600 Indian patients suggested that only polymorphisms existing simultaneously in all three of the genes studied constituted a CRC risk factor [29]. In our study, the presence of all three polymorphisms simultaneously, i.e. *GSTM1 null*, *GSTT1 null*, and *GSTP1 Ile_105_Val* (homo- or heterozygous), did not differ (*p* = 0.99) between patients (7.6%) and controls (6.8%).

In conclusion, ours was the first analysis of *GST* gene polymorphisms and CRC risk in Polish non-smokers. Mutation frequencies observed in healthy individuals were consistent with other studies conducted in Europe. *GSTT1* polymorphism correlated with higher tumor grade in CRC patients (*p* = 0.035), and the *GSTM1 null/null* genotype was associated with more frequent metastasis to lymph nodes (pN classification, *p* = 0.036). However, most *GST* gene polymorphism differences between patient and control groups were not significant in our cohort, which was relatively small due to the exclusion of current and former smokers (some 60% of CRC patients assessed were excluded from our study for this reason). Therefore, our study will be continued in cooperation with other oncological centers in Poland in a larger cohort. Additionally, further subdivision of patient groups with respect to red meat consumption and physical activity levels could reveal correlations between coexisting genetic and environmental factors and CRC risk.

### Limitations and clinical implications of the study

The aim of the study was to investigate the relationship between polymorphism of GST genes and colorectal cancer risk. Confirmation of the influence of the studied polymorphisms on the susceptibility of CRC development would allow to extend the applied tests with an additional risk factor of genetic predisposition to the development of the disease. The recruitment of patients was significantly prolonged due to the exclusion of current or former smokers, the pool of patients was severely limited. About 60% of colorectal cancer was rejected due to this exclusion criterion.

## MATERIALS AND METHODS

### Population

This study was conducted from 2014–2017 in two clinical centers in Kielce, Poland: the Kielce Provincial Hospital and the Holy Cross Cancer Centre. Patient inclusion and exclusion criteria are presented in Table 4. Only non-smoking patients were included in the study due to correlations between tobacco smoke and *GST* gene polymorphisms [11, 12, 13]. Results from studies that do not separate smokers and non-smokers sometimes suggest an increased risk of CRC in patients with *GST* polymorphisms. However, such studies cannot determine whether *GST* mutations that reduce glutathione transferase activities contribute to carcinogenesis itself or are consequences of tobacco smoke, which is a well-known carcinogen. Our study included 197 patients with colorectal adenocarcinoma confirmed by pathological diagnosis of specimens collected during colonoscopy or surgery. The control group consisted of 104 patients without cancer, as confirmed by an endoscopic and/or histopathological examination. All patients signed written consent forms for genetic testing of *GST* genes. Clinical data and blood samples were collected (test tubes with EDTA provided by Sarsted, Warszawska 25, 05-082 Stare Babice, Poland ). Coded samples were frozen at −20° C for genetic testing. The study was approved on June 3, 2013 by the local Bioethics Commission (No. 5/2013) on the basis of an application with an exact description of the procedure.

**Table 4:** Inclusion/exclusion criteria

	Patients	Controls
Inclusion criteria	- Age 35–85 - CRC as confirmed by histopathological examination	- Age 35–85 - No colon tumors or polyps as confirmed by colonoscopy
Exclusion criteria	- smoking tobacco currently and/or previously - genetic predisposition to CRC (FAP, HNPCC) - CRC in family history	- smoking tobacco currently and/or previously - genetic predisposition to CRC (FAP, HNPCC) - CRC in patient and/or family history

### Genotyping

Peripheral blood leukocytes were used for genetic testing. Genomic DNA was isolated from blood samples using the Genomic Micro AX Blood Gravity kit from AA Biotechnology. Purity and concentration of isolated DNA were evaluated spectrophotometrically at 260 nm and 280 nm (Nanodrop 2000, Thermo Fisher Scientific). Analysis of the *GSTP1* gene rs1695 polymorphism was conducted using TaqMan qPCR SNP endpoint genotyping (Assay ID C_3237198_20). Deletions in *GSTT1* (Assay ID Hs00010004_cn) and *GSTM1* (Assay ID Hs02575461_cn) were analyzed using the qPCR relative quantification method with *TERT* as the control gene. In all cases, the Light Cycler 96 instrument and TaqMan primer/probe kit (produced by Life Technology) were used. PCR amplification using approximately 10 ng of genomic DNA was performed with an initial step of 95° C for 10 min followed by 50 cycles of 95° C for 15 s and 60° C for 90 s.

### Statistical analysis

Categorical data were expressed as number and percentage distributions. Chi-square test or Fisher's exact test were applied to compare proportions. A two-tailed *p* < 0.05 was considered statistically significant. All statistical analyses were performed using R (version 3.1.2; The R Foundation for Statistical Computing, Vienna, Austria) and Statistica (StatSoft, Inc. 2014, version 12).
